# Novel agents for advanced pancreatic cancer

**DOI:** 10.18632/oncotarget.3999

**Published:** 2015-06-03

**Authors:** Akintunde Akinleye, Chaitanya Iragavarapu, Muhammad Furqan, Shundong Cang, Delong Liu

**Affiliations:** ^1^ Division of Hematology/Oncology, Department of Medicine, New York Medical College, Valhalla, New York, United States; ^2^ Division of Hematology/Oncology, Department of Medicine, University of Iowa, Iowa City, Iowa, United States; ^3^ Department of Oncology, Henan Province People's Hospital, Zhengzhou University, Zhengzhou, China; ^4^ Department of Oncology, Henan Cancer Hospital and the Affiliated Cancer Hospital of Zhengzhou University, Zhengzhou, China

**Keywords:** pancreatic cancer, IGF-1R

## Abstract

Pancreatic cancer is relatively insensitive to conventional chemotherapy. Therefore, novel agents targeting dysregulated pathways (MAPK/ERK, EGFR, TGF-β, HEDGEHOG, NOTCH, IGF, PARP, PI3K/AKT, RAS, and Src) are being explored in clinical trials as monotherapy or in combination with cytotoxic chemotherapy. This review summarizes the most recent advances with the targeted therapies in the treatment of patients with advanced pancreatic cancer.

## INTRODUCTION

Pancreatic ductal adenocarcinoma (PDAC), otherwise known as pancreatic carcinoma or pancreatic cancer, is a highly aggressive malignancy characterized by local and vascular invasion, extensive regional lymph node metastasis, and distant metastases [1]. PDAC is the ninth most common malignancy in western countries, but represents the fourth leading cause of cancer related death [2, 3]. Approximately 80% of patients have either unresectable locally advanced or metastatic disease at the time of diagnosis and three-quarter of those who undergo curative surgical resection develop recurrent disease [4–7]. As such, pancreatic cancer is considered to be a systemic disease in the majority of patients at the time of diagnosis.

Unfortunately, PDAC typically demonstrates innate resistance to conventional chemotherapeutics, due in part to multiple molecular aberrations, dense desmoplastic reaction, poor angiogenesis and tumor microenvironment hypoxia [8–10]. Responses are often short-lived and prognosis is dismal. Primary resection with subsequent adjuvant chemotherapy yields a median survival of 20–22 months in resectable disease [6, 7, 11, 12]. With current chemotherapy regimens, the median survival for patients with unresectable/metastatic tumors is 9–11 months [13–15].

A better understanding of the molecular basis of pancreatic carcinogenesis has led to the development of strategies targeting dysregulated signaling pathways implicated in the development and progression of this devastating cancer. These targeted therapies include small-molecule inhibitors of signaling proteins such as Hedgehog, MEK, RAS, and SRC [16–21]; as well as cell-membrane proteins such as EGFR [22]. This review discusses new targeted therapies that have progressed from preclinical studies into clinical trials for the treatment of patients with advanced pancreatic cancer either as monotherapy or in combination with cytotoxic agents in an attempt to achieve better responses and improve survival.

## CONVENTIONAL APPROACHES

In newly diagnosed patients with advanced unresectable /metastatic pancreatic cancer, gemcitabine- and fluoropyrimidine- based chemotherapy have been established as the preferred initial treatment options for most patients with the goal of prolonging survival and improving the quality of life. Specifically, gemcitabine plus nab-paclitaxel and FOLFIRINOX (combination of 5-fluorouracil, folinic acid, irinotecan and oxaliplatin) demonstrated high response rates and modest improvement in overall survival [14, 15]. In contrast, the combination of gemcitabine with other cytotoxic agents including oxaliplatin, irinotecan, cisplatin, fluorouracil/leucovorin, pemetrexed or capecitabine failed to show a survival benefit over gemcitabine alone in prospective phase II/III studies (Table 1) [23–29]. Similarly, gemcitabine with monoclonal antibodies (e.g bevacizumab, cetuximab, ganitumab) did not reveal improvement in survival when compared to gemcitabine alone in phase III CALGB, SWOG, and GAMMA trials (Table 2) [30–32]. Recent trials employing gemcitabine plus small molecule inhibitors such as sorafenib, tipifarnib, rigosertib, trametinib, aflibercept, sunitinib, everolimus, or axitinib failed to show better survival in comparison with gemcitabine monotherapy in randomized placebo-controlled phase II/III studies in previously untreated patients with advanced pancreatic cancer (Table 3) [33–39]. Nevertheless, Moore et al reported that gemcitabine plus erlotinib conferred significant survival advantage with improved overall survival (OS) and progression free survival (PFS) rates [40]. These findings underscore further development of targeted agents in the treatment of patients with advanced unresectable and metastatic pancreatic adenocarcinoma.

**Table 1:** Phase III trials of gemcitabine containing regimens in advanced pancreatic cancer

Trial	Regimen	Primary endpoint	Reference
MPACT	gemcitabine + nab-paclitaxel vs. gemcitabine	mOS: 8.5 vs. 6.7 months (*p* < 0.001)	[15]
GIP-1	gemcitabine + cisplatin vs. gemcitabine	mOS: 8.3 vs. 7.2 months (*p* = 0.38)	[23]
GEM-CAP	gemcitabine + capecitabine vs. gemcitabine	mOS: 7.1 vs. 6.2 months (*p* = 0.08)	[24]
E6201	gemcitabine + oxaliplatin vs. gemcitabine	mOS: 5.7 vs. 4.9 months (*p* = 0.22)	[25]
NCT00023972	gemcitabine + exatecan vs. gemcitabine	mOS: 6.7 vs. 6.2 months (*p* = 0.52)	[26]
Stathopoulos et al.	gemcitabine + irinotecan vs. gemcitabine	mOS: 6.4 vs. 6.5 months (*p* = 0.957)	[27]
NCT00035035	gemcitabine + pemetrexed vs. gemcitabine	mOS: 6.2 vs. 6.3 months (*p* = 0.8477)	[28]
E2297	gemcitabine + 5-FU vs. gemcitabine	mOS: 6.7 vs. 5.4 months (*p* = 0.09)	[29]

Abbreviations: 5-FU – 5-fluorouracil; mOS – median overall survival.

**Table 2:** Phase III trials of gemcitabine with biologics in advanced pancreatic cancer

Trial	Regimen	Primary endpoint	Reference
SWOG S0205	gemcitabine + cetuximab vs. gemcitabine	mOS: 6.3 vs. 5.9 months (*p* = 0.23)	[30]
CALGB 80303	gemcitabine + bevacizumab vs. gemcitabine	mOS: 5.8 vs. 5.9 months (*p* = 0.95)	[31]
GAMMA	gemcitabine + ganitumab vs. gemcitabine	mOS: 7.1 vs. 7.0 months (*p* = 0.397)	[32]

Abbreviation: mOS: median overall survival.

**Table 3:** Phase II/III trials of gemcitabine with small molecule inhibitors in advanced pancreatic cancer

Trial	Regimen	Primary endpoint	Reference
BAYPAN	gemcitabine + sorafenib vs. gemcitabine	mPFS: 3.8 vs. 5.7months (*p* = 0.902)	[33]
NCT00471146	gemcitabine + axitinib vs. gemcitabine	mOS: 8.5 vs. 8.3 months (*p* = 0.5436)	[34]
CESAR	gemcitabine + sunitinib vs. gemcitabine	mPFS: 11.6 vs. 13.3 weeks (*p* = 0.74)	[35]
NCT00574275	gemcitabine + aflibercept vs. gemcitabine	mOS: 6.5 vs. 7.8 months (*p* = 0.2034)	[36]
NCT00005648	gemcitabine + tipifarnib vs. gemcitabine	mOS: 193 vs. 182 days (*p* = 0.75)	[37]
NCT00409292	gemcitabine + everolimus	mOS: 4.5months	[38]
NCT01231581	gemcitabine + trametinib vs. gemcitabine	mOS: 8.4 vs. 6.7 months (*p* = 0.453)	[39]
NCIC CTG PA.3	gemcitabine + erlotinib vs. gemcitabine	mOS: 6.24 vs. 5.91 months (*p* = 0.038)	[40]

Abbreviations: mOS – median overall survival; mPFS – median progression-free survival.

## NOVEL MONOCLONAL ANTIBODIES

### Tigatuzumab (CS-1008)

Tigatuzumab, also known as CS-1008, is an intravenously bioavailable, humanized murine IgG1 monoclonal antibody with a molecular weight of 144.6kDA, and composed of the complementarity determining region (CDR) of the murine monoclonal antibody TRA-8 and the variable region framework and constant regions of human immunoglobulin IgG-1mAb58′CL [41, 42]. The antibody demonstrates potent agonist property against TRAIL (tumor necrosis factor-related apoptosis-inducing ligand) receptor 2 (TR-2)/death receptor 5 (DR5). This antibody induces tumor cell apoptosis and growth inhibition by triggering both extrinsic and intrinsic apoptotic, caspase-mediated signaling pathways [43, 44]. *In vitro* studies showed that tigatuzumab induces selective, dose-dependent cytotoxicity in several human pancreatic carcinoma cell lines including MIA PaCa-2 [41, 45, 46]. Tigatuzumab did not induce cell death in human primary hepatocytes. In xenografted MIA PaCa-2 mouse model of human pancreatic carcinoma, tigatuzumab dosed at 0.3 and 3 mg/kg in combination with gemcitabine at 400 mg/kg substantially inhibited tumor growth with complete tumor regression noted in two of 10 mice treated with the higher tigatuzumab dose [41].

In an initial, multi-institutional, open-label, phase I dose-escalation study, tigatuzumab at dose levels of 1, 2, 4, and 8 mg/kg was administered weekly by intravenous infusion to 17 patients with relapsed or refractory carcinomas and lymphoma [47]. After at least 2 cycles of treatment, tigatuzumab was found to be safe, well-tolerated, with no dose-limiting toxicity (DLT), and the maximal tolerated dose (MTD) was not reached. The most common adverse events were nausea, vomiting, fatigue, pyrexia, anemia, and cough. No serious treatment-related toxicities observed [47]. Stable disease was achieved in approximately 41% of patients for a prolonged period of time [47]. These results prompted a phase II trial to evaluate the efficacy of tigatuzumab administered in combination with gemcitabine to chemotherapy-naive patients diagnosed with unresectable or metastatic pancreatic cancer [48]. Sixty-five patients, predominantly Caucasians with median age of 60.6 years, received tigatuzumab intravenously on days 1, 8, 15, and 21 (8 mg/kg loading dose followed by 3 mg/kg per week) and intravenous gemcitabine on days 1, 8, and 15 (1000 mg/m2) until disease progression or unacceptable toxicity. Tolerability profile was acceptable as the most common toxicities were grade 1 or 2 nausea, fatigue, abdominal pain, constipation, and fever after a median duration of treatment of approximately 18 weeks. No new treatment-emergent adverse events were seen [47, 48]. Of 61 patients evaluated for efficacy, the overall response rate (ORR) was 13.1% with a median duration of response of 309 days. The primary endpoint, PFS at 16 weeks, was 53%, and the median PFS and OS were 3.9 months and 8.2 months respectively [48]. Tigatuzumab combined with gemcitabine was well tolerated and may improve survival outcomes in patients with unresectable or metastatic pancreatic cancer. Tigatuzumab has now entered phase II clinical trials for the treatment of patients with a variety of solid neoplasms including pancreatic cancer, non-small cell lung cancer (NSCLC), hepatocellular carcinoma, and ovarian cancer [NCT00521404, NCT00991796, NCT01033240, NCT00945191].

### Dalotuzumab (MK-0646)

**Figure 1:**
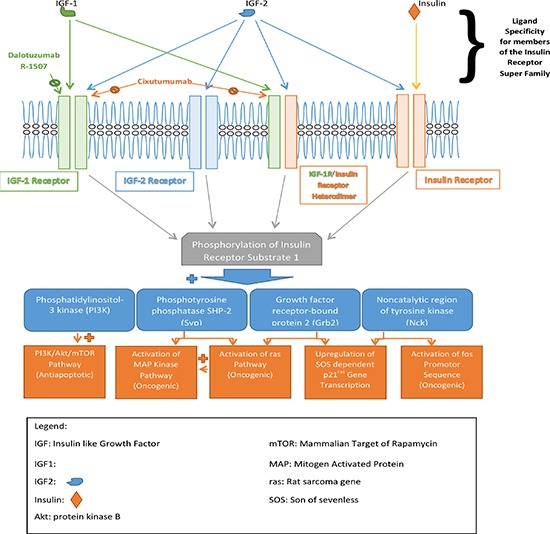
Signaling pathways for insulin and insulin-like growth factors Three growth factors, insulin, insulin-like 1, and 2 signal through four major receptors—homodimeric insulin-like growth factor 1 receptor (IGF-1R), homodimeric insulin-like growth factor 2 receptor (IGF-2R), heterodimeric insulin receptor (IR) /IGF-1R, and homodimeric IR. There pathways contribute to diverse cellular functions, including proliferation, survival, apoptosis, and cell metabolism. The corresponding ligands activating these receptors are highlighted. The diverse downstream signaling proteins are indicated. Dalotuzumab, R-1507, and cixutumumab are the agents in development targeting IGF receptors.

The insulin growth factor-1 receptor (IGF-1R) is a homodimeric transmembrane tyrosine kinase receptor. It.is over-expressed on the surface of several human malignancies including pancreatic tumor cells [49–52]. When activated by its ligands (IGF-I and -II), the receptor sends signals that exert antiapoptotic effects and confers increased tumor growth, resistance to chemotherapeutics, and motility of cancer cells via multiple signaling cascades (Figure 1) [53, 54]. Dalotuzumab is a novel, recombinant humanized IgG1 monoclonal antagonist antibody that binds to IGF-1R with high affinity (K_d_ = 1 nmol/L). This induces receptor internalization and degradation, and inhibits IGF-I- and IGF-II- mediated pancreatic cancer growth and metastasis [55–57]. In preclinical studies, dalotuzumab (also known as MK-0646 or h7C10) enhances gemcitabine-induced apoptosis and inhibits signaling pathways that confer increased cellular proliferation, survival and drug resistance in pancreatic cancer [56, 58]. The first-in-human study of dalotuzumab showed the agent was generally well-tolerated, exhibited positive pharmacodynamic effects, and favorable clinical activity in patients with advanced solid cancers [59]. The most common adverse event was hyperglycemia. Subsequently, a phase I/II study evaluated dalotuzumab in combination regimens. Jayle and colleagues showed that dalotuzumab (5–10 mg/kg weekly) plus gemcitabine (1000 mg/m2 weekly) or gemcitabine (100 mg/m2 weekly) and erlotinib (100 mg daily) showed favorable toxicity profile in previously untreated patients with metastatic pancreatic cancer [60]. Serious adverse events included hyperglycemia, hepatic toxicity, and cytopenia. Early efficacy data showed higher partial response (PR) in the dalotuzumab plus gemcitabine and erlotinib arm (25% versus 20%). Additional clinical data clarifying the OS benefit of this targeted agent are expected from ongoing clinical trials.

### Conatumumab (AMG-655)

Conatumumab (AMG-655) is a fully human IgG1 monoclonal antibody with antineoplastic activity mediated by agonist effect against the extracellular domain of human TR-2) /DR5 [61]. Conatumumab binds to and activates TR-2 /DR5, leading to activation of downstream caspase cascades and tumor cell apoptosis. TR-2 is highly expressed by pancreatic tumor cells than normal pancreatic tissue. The TR-2 high expression makes it an attractive, druggable target for cancer therapeutics [62]. Treatment with conatumumab alone or in combination with gemcitabine demonstrated potent antitumor activity against pancreatic tumor xenograft [61]. The safety and preliminary clinical activity of conatumumab in combination with gemcitabine have been evaluated in a phase IB multicenter trial of 13 previously-untreated patients with advanced pancreatic cancer [63]. Participants received conatumumab 3 or 10 mg/kg IV day 1 and 15, and gemcitabine 1000 mg/m2 IV on day 1, 8, and 15 of every 28-day cycle. After a median treatment of 6 cycles, the combination appeared tolerable and no DLTs were observed. Thrombocytopenia, abdominal pain, and neutropenia were the most common serious adverse events. Preliminary efficacy data showed a PR of 31%, median PFS of 5.3 months, and a 6-month OS rate of 76% [63]. Given these data, conatumumab combined with gemcitabine was advanced to a placebo-controlled, randomized phase II clinical testing [64]. No new safety signal emerged after median treatment of 4 cycles. The disease control rate was 65% in the combination arm versus 40% for the placebo arm, with an improved 6-month OS rate of 59% versus and 50% respectively [64]. Additional investigation into this combination in patients with advanced/metastatic pancreatic cancer is warranted.

### Cixutumumab (IMC-A12)

Cixutumumab, also known as IMC-A12, is a potent, highly selective, fully human IgG1 monoclonal blocking antibody with dual, subnanomolar inhibitory activity (IC_50_ = 0.6–1 nM) against IGF-1R homodimers as well as heterodimeric insulin receptor /IGF-1R in tumor cells (Figure 1) [65, 66]. Cixutumumab lacks antibody-dependent cellular cytotoxicity (ADCC). Rather, it selectively binds to IGF-1R or IR/IGF-1R with high affinity (K_d_ = 0.04 nM), triggers internalization and degradation of the receptor, and indirectly blocks both IGF-I and -II mediated ERK-MAPK (proliferative) as well as the PI3K-AKT-mTOR (survival) signaling pathway activation [66]. Cixutumumab exhibited broad anti-mitogenic activity on multiple tumor cell lines including breast cancer line (T47D), pancreatic cancer line (BxPC-3), and multiple myeloma cell line (RPMI-8226) [66]. In BxPC-3 pancreatic cancer xenograft models, cixutumumab induced tumor shrinkage by eighty percent at a dose of 1mg every three days [66].

Infusion reactions, anemia, rash, pruritus, dizziness, and fatigue were the common side-effects observed in the first phase I dose escalation study of 16 patients with relapsed/refractory solid neoplasms including breast, bladder, pancreatic, endometrial, hepatocellular carcinoma, and phaechromocytoma [67]. The DLT was hyperglycemia. Though no objective response was observed, nine patients experienced stable disease lasting for more than 6 weeks [67]. This preliminary evidence of activity of cixutumumab has prompted multiple early phase trials in advanced solid tumors. In a recent report of a phase IB/II SWOG S0727study, administration of cixutumumab 6 mg/kg/week intravenously in combination with erlotinib 100 mg/day orally, and gemcitabine 1000 mg/m2 intravenously on days 1, 8, and 15 of a 28-day cycle demonstrated tolerable toxicity profile in chemotherapy-naïve patients with advanced/metastatic pancreatic cancer [68]. The most common serious adverse effects were transaminitis, hyperglycemia, fatigue, and cytopenia. When compared with the control arm (erlotinib plus gemcitabine), the addition of cixutumumab to erlotinib and gemcitabine did not lead to longer PFS or OS. The median PFS and OS were 3.6 and 7.0 months respectively on the cixutumumab arm, and 3.6 and 6.7 months respectively on the control arm [68].

At this time, it is unclear how much, if any, benefit this specific agent would add to that achieved with conventional approaches. Evaluation of cixutumumab with other active agents in pancreatic cancer is warranted in future trials to determine whether or not this antibody will produce clinically, meaningful benefit in patients with PDAC.

### R-1507

Like dalotuzumab, R-1507 is a humanized IgG1 monoclonal antibody with potent, highly selective, antagonist activity against the extracellular domain of IGF-1R characterized by intracellular internalization and degradation of the receptor [69]. The antibody demonstrates antitumor activity against pancreatic cell lines and delays tumor growth in xenograft models [70]. Preclinical studies by Kawanami and colleagues showed that R-1507 interacted synergistically with gemcitabine and/or metformin to inhibit proliferation and growth in human pancreatic ductal adenocarcinoma cell lines SUIT-2 and MIAPaCa-2 [71]. Treatment with R-1507 and everolimus, an mTOR inhibitor, demonstrated substantial anti-proliferative activity against pancreatic cell line BxPC-3 [72]. The agent was well-tolerated up to a dose of 9 mg/kg weekly in a phase I trial [73]. No DLTs were observed and the most common drug-related adverse effects were fatigue and nausea. Further investigations are warranted to establish whether IGF-1R inhibition with R-1507 alone or in combination with cytotoxic chemotherapy will improve outcomes in patients with advanced pancreatic cancer.

### AGS-1C4D4

AGS-1C4D4 is a fully human IgG1 monoclonal antibody directed against the human prostate stem cell antigen (PSCA) with potential anti-neoplastic activity. The mechanism of action is designed to selectively bind to the extracellular domain of PSCA and triggers complement-dependent cell lysis (CDC) and ADCC in tumor cells expressing PSCA [74]. PSCA is a glycosylphosphatidylinositol (GPI)-linked cell surface protein. It is overexpressed on the cell surfaces of a variety of cancer cell types, including pancreatic cancer, and may play a key role in cell proliferation, invasion, and survival [75, 76]. Targeting PSCA has been shown as a promising anti-tumor strategy in pancreatic cancers in preclinical models [77, 78].

In phase I studies, the compound demonstrated acceptable toxicity profile with no DLTs at 48 mg/kg loading dose followed by 24 mg/kg every 3 weeks intravenously [74, 79], and this dose was carried to a randomized phase II trial in patients with advanced/metastatic pancreatic cancer [80]. In this multicenter study, previously untreated, metastatic pancreatic adenocarcinoma patients were randomly assigned 1:2 to gemcitabine (1000 mg/m2) or gemcitabine plus AGS-1C4D4 antibody [80]. The trial met its primary endpoint. The 6-month survival rate was significantly improved in the combination therapy arm compared to gemcitabine alone arm (60.9% versus 44.4%; *p* = 0.03), while the median OS were 7.6 months and 5.5 months in the two arms, respectively [80]. Another phase II trial is investigating the activity and safety of this regimen in the second-line therapy for chemotherapy-refractory patients with advanced/metastatic pancreatic cancer [NCT01608711].

### Tarextumab (OMP-59R5)

Tarextumab (formerly OMP-59R5) is a novel, highly selective, fully human IgG2 monoclonal blocking antibody that binds to and prevents signaling through both the Notch2 and Notch3 receptors [81]. Notch signaling plays a critical role in pancreatic cancer transformation, tumor progression, and chemotherapy resistance. In preclinical models, tarextumab exhibited substantial tumor regressions in Notch3-expressing human pancreatic cancer xenografts when combined with nab-paclitaxel and gemcitabine via inhibition of cancer stem cell growth, promotion of cell differentiation, as well as disruption of tumor angiogenesis [81]. Although the anti-NOTCH agent (RO4929097) failed to improve survival [82], final data from a phase IB trial demonstrated encouraging clinical activity of tarextumab in combination with nab-paclitaxel and gemcitabine in treatment naïve pancreatic cancer patients [81, 83]. The combination was well-tolerated and no DLTs occurred. Frequently reported adverse effects were cytopenia, fatigue, GI toxicities, peripheral neuropathy, and alopecia. A phase II ALPINE study of this combination is underway [NCT01647828].

## RADIOIMMUNOCONJUGATE

### ^90^Yttrium-clivatuzumab tetraxetan (90Y-hPAM4)

Yttrium (^90^Y) clivatuzumab tetraxetan (also known as hPAM4-Cide) is a radioimmunoconjugate composed of fully humanized monoclonal antibody HuPAM4, directed against the pancreatic cancer antigen mucin-1(MUC-1), that is conjugated to the chelating agent tetra-azacyclododecanetetra-acetic acid (DOTA), and radiolabeled with the beta-emitting radioisotope Yttrium^90^ [84]. MUC-1is a cell membrane surface glycoprotein with extensive O-linked glycosylation of its extracellular domain [85]. It is overexpressed in more than 85% of pancreatic adenocarcinomas and absent in normal pancreas, making it an attractive target for anti-cancer therapeutics [86–88]. Aberrant activation of the MUC-1-dependent pathways has been implicated in initiation and maintenance of malignant phenotype. Increased expression enhances beta-catenin mediated cancer invasion, and promotes both p53- and PI3K- AKT-dependent cell survival [89–91]. Clivatuzumab tetraxetan demonstrates ADCC. The compound selectively binds to tumor cells expressing MUC-1, undergoes internalization, and delivers high cytotoxic dose of beta radiation to the tumor cells [92, 93]. In athymic nude mice bearing CaPan1 human pancreatic cancer xenografts, clivatuzumab alone or in combination with gemcitabine exhibited substantial antitumor response [94–97].

A phase I dose-escalation trial of clivatuzumab teraxetan enrolled 21 patients with advanced pancreatic cancer. These patients were treated with single dose of 15, 20, or 25 mCi/m2. The radioimmunoconjugate was well-tolerated. DLTs included neutropenia and thrombocytopenia, which occurred at 25 mCi/m2. Therefore, 20 mCi/m2 was established as the MTD and chosen for further studies [98]. Notably, 2 patients developed human anti-human antibody (HAHA). Of twenty patients evaluated for response at week 4 after treatment, there were three PR and four with stable disease (SD) [98]. When clivatuzumab was administered at weekly fractionated doses together with low dose gemcitabine, no new treatment-related toxicities were noted [84]. The disease control rate was higher at 58% (6 PR, 16 SD) presumably due to the radiosensitizing effect of gemcitabine.

These encouraging results led to initiation of phase II/III clinical trials of clivatuzumab in advanced pancreatic cancer. Final results of a multicenter phase IB study of 58 heavily-pretreated patients with pancreatic cancer were recently reported at the 2014 ASCO Annual Meeting [99]. In arm A, twenty-nine patients received the combination of fractionated doses of clivatuzumab tetraxetan once-a-week for 3 weeks with gemcitabine given weekly for 4 weeks while 29 patients in arm B were treated with 4 doses of clivatuzumab tetraxetan alone. The treatment cycle was repeated every 4 weeks until unacceptable toxicity or disease progression or patient withdrawal. The median OS of patients who received combination therapy (7.9 months) was significantly longer than that of patients who received antibody alone (3.4 months) [*P* = 0.004] [99]. Given these favorable results, a phase III PANCRIT-1 registration trial has been initiated to confirm these findings.

## SMALL MOLECULE INHIBITORS

### Vismodegib (GDC-0449)

Vismodegib (GDC-0449, HhAntag-691) is an orally administered, specific, irreversible, and highly potent hedgehog pathway (Hh) inhibitor approved by the FDA in January 2012 for treatment of patients with unresectable locally advanced/metastatic basal cell carcinoma of the skin [100, 101]. The Hh pathway is activated by binding of Hh ligands (Sonic, Desert, Indian) to the transmembrane G-protein-coupled receptor (Patched), which in turn releases the suppression of signal transducing protein (Smoothened) that triggers a cascade of events resulting in expression of Hh-responsive genes [102]. Uncontrolled activation of the Hedgehog (Hh) signaling pathway has emerged as a central player in neoplastic transformation, tumor growth and cancer survival in a growing number of hematologic and solid malignancies including the skin, breast, lung, liver, and pancreatic carcinomas along with colon and brain tumors [103–108]. The components of Sonic Hh signaling pathway, including the ligand (SHh), the signaling molecules (Patched-1, Patched-2 and Smoothened) and effectors (Gli1, and Gli2) are aberrantly expressed in human pancreatic cancer cell lines and pancreatic cancer stem cells [109–111]. Vismodegib induces cell apoptosis and inhibits survival in dose-dependent manner in pancreatic cell lines by selectively binding to and downregulating the expression of Smoothened, Patched-1, and Patched-2 [112, 113]. Similarly, vismodegib inhibited the expression of transcription factor Gli1 and Gli2 [112].

Vismodegib has been assessed in a multi-center, placebo-controlled, phase IB/randomized phase II study of previously untreated patients with metastatic pancreatic cancer [114]. One hundred and six patients were randomized in 1:1 to gemcitabine (1000 mg/m2 over 30 minutes on days 1, 8, 15), every 28 days plus either placebo (GP) or vismodegib (150 mg PO daily) (GV). Toxicity between the 2 groups was similar. The most serious adverse events were cytopenia, hyponatremia, fatigue, and hyperglycemia [114]. Adding vismodegib to gemcitabine chemotherapy as frontline therapy yielded disappointing results. No improvement in median PFS (4.0/2.5 months) and median OS (6.9/6.1 months) when compared with gemcitabine alone, although 22 patients on the GP crossed over to the GV at progression, which could have complicated the outcome [114]. Interim results from an ongoing open label phase II trial of untreated pancreatic cancer patients showed that vismodegib (150mg PO daily) combined with gemcitabine (1000 mg/m2) plus nab-paclitaxel (125 mg/m2) on days 1, 8 and 15 of every 28 days cycle demonstrated acceptable toxicity profile [115]. Of forty-nine patients evaluated for response, the ORR was 43%. Data analysis showed a median PFS of 5.5 months and OS of 10 months [115].

### Masitinib (AB1010)

Masitinib (AB1010) is a potent, ATP competitive, multi-tyrosine kinase inhibitor with nanomolar activity (≤ 500 nM) [116] and potential antineoplastic activity. A benzamide derivative, masitinib selectively binds to and inhibits both the wild-type and mutated forms of the stem cell factor receptor (c-Kit; SCFR); platelet-derived growth factor receptor (PDGFR); and fibroblast growth factor receptor 3 (FGFR3) [116]. The compound enhances the antiproliferative effects of gemcitabine in gemcitabine-refractory human pancreatic cell lines Mia Paca2 and Panc1 by downregulating Wnt/beta-catenin signaling pathway [117]. Substantial clinical progress has been seen with masitinib. In a phase I dose-escalation study conducted in patients with advanced and/or metastatic pancreatic cancer, the MTD was not reached but a dose of 12 mg/kg/day was found to be safe for the treatment of patients with solid cancers [118]. In a phase II trial in combination with gemcitabine, masitinib demonstrated substantial clinical activity in treatment naïve patients with advanced pancreatic cancer [119], resulting in its advancement to phase III registration study. This trial met its primary endpoint of median time-to-progression (TTP) (6.4 months) which was well beyond the threshold (2.1 months) set by the investigators [119]. The results of the first prospective, international, randomized, double-blinded clinical trial of masitinib plus gemcitabine as first line treatment in patients with advanced pancreatic cancer were presented at the 2013 American Society of Clinical Oncology (ASCO) meeting. Three hundred and fourty eight patients were randomized to receive either masitinib (9 mg/kg/day) in combination with gemcitabine (1000 mg/m2/weekly) or placebo plus gemcitabine with overall survival (OS) being the primary end point [120]. A gene expression profiling assay of whole blood samples was also conducted before initiation of treatment to identify genetic expression patterns predictive of overall survival and/or treatment benefit. Overall, no difference in OS was demonstrated (HR = 0.90; 95% CI, 0.71–1.14; *p* = 0.74). However, masitinib in combination with gemcitabine significantly extended median OS in two independent patient populations [120]. Firstly, median OS was significantly prolonged in patients with pain at baseline (defined as a VAS score > 20 mm on a 100 mm scale) from 5.4 months in the placebo arm to 8.1 months in the masitinib arm (*p* = 0.010). In another separate cohort analysis of patients with a specific deleterious genomic biomarker (GBM) indicative of aggressive disease, the median OS was superior with combination of masitinib plus gemcitabine compared with gemcitabine plus placebo (11.0 versus 5.0 months, respectively; *p* = 0.000038) [120].

### Selumetinib (AZD-6244, ARRY-142886)

Selumetinib (also known as AZD-6244 or ARRY-142886) is an orally bioavailable, non-ATP competitive, highly selective MEK 1/2 inhibitor with potential anti-neoplastic activity at nanomolar concentration (IC_50_ of 14 nm) [17, 121]. In murine BxPC3 pancreatic tumor xenograft models, selumetinib significantly inhibited tumor growth [121], and its antitumor activity correlated with substantial decrease in phosphorylated ERK1/2 levels. A phase I dose escalation study of 57 patients with advanced cancers including pancreatic carcinoma identified 100mg BID as safe and well-tolerated [122]. Rash, diarrhea and hypoxia were reported as the major DLTs. Results of a recently published phase II trial showed no difference in overall survival between selumetinib and capecitabine as second-line treatment in 70 patients with advanced pancreatic cancer who had been pretreated with a gemcitabine-based regimen [20]. The median OS was 5.4 months in the selumetinib group versus 5.0 months in the capecitabine group (*p* = 0.92) [20]. Dual targeting of MEK/EGFR signaling with selumetinib and erlotinib in 46 previously-treated patients with advanced/metastatic pancreatic cancer demonstrated a disease control rate of 51%. The estimated median PFS and OS were 2.6 and 7.5 months respectively [123]. Additional clinical trials are currently underway to further explore this targeted agent in combination strategies [NCT01658943, NCT01061749].

### Saracatinib (AZD-0530)

A quinazolinamine derivative, saracatinib (formerly AZD-0530) is an orally bioavailable, dual-specific inhibitor of Src and Abl tyrosine kinases with antitumor activity at nanomolar concentrations (IC_50_ = 4–10 nM) [124]. In preclinical models of pancreatic cancer, saracatinib showed great antitumor activity in orthotopic ASPC-1xenograft mice through inhibition of Src phosphorylation and induction of cell cycle arrest at G1/S [124].

In first-line setting, phase I/II trial by Renouf et al. [125] evaluated the combination of saracatinib (175 mg PO daily) and gemcitabine (1000 mg/m2) in patients with advanced pancreatic cancer and showed the combination was safe and generally well-tolerated. The phase II part of the trial did not meet its primary endpoint of objective tumor response (ORR) plus stable disease ≥ 4 months, and was closed to further accrual [125].

### Pimasertib (AS-703026, MSC-1936369B)

Pimasertib, also known as AS703026, MSC1936369B, is a highly potent, ATP noncompetitive, second generation small molecule inhibitor of MEK1 and MEK2 [126]. It exhibited potent antitumor activity by selectively binding to the allosteric site on MEK1/2; induced G0-G1 cell cycle arrest via downregulation of pERK1/2; and triggered apoptosis by caspase-3 and PARP cleavage [127]. Interim safety data from an ongoing open label phase II trial showed that pimasertib was safe and well-tolerated when combined with gemcitabine in chemotherapy naïve patients with advanced pancreatic cancer [128]. The DLTs were grade 3 confusion with ataxia and disorientation, and grade 4 suicidal ideation. Asthenia, ocular disturbance, skin rash, GI toxicities, and cytopenias were the most common adverse events [128]. Further clinical data are awaited.

### Refametinib (RDEA119, BAY 869766)

Refametinib is another MEK inhibitor that has progressed to early phase clinical testing in patients with advanced/metastatic pancreatic cancer. A cyclopropane-1-sulfonamide derivative, refametinib inhibits cell proliferation in several tumor cell lines including BxPC3 human pancreatic cell line [129]. The compound alone or in combination with rapamycin, an mTOR inhibitor, showed significant growth inhibition in murine xenograft models of human pancreatic cancer cells OCIP19, 21, and 23 mediated by cell cycle arrest predominantly in G1 phase [130]. The combination of refametinib and gemcitabine as first-line treatment of sixty patients with advanced pancreatic cancer showed encouraging results in a single-arm, open label, phase IIA clinical trial with stable disease and partial response shown in 38% and 35%, respectively [131]. The compound demonstrated acceptable safety profile with most common toxicities being cytopenia, transaminitis, hypertension, rash and fatigue [131].

### Galunisertib (LY-2157299)

Galunisertib, also known as LY-2157299, is a novel selective small molecule inhibitor of transforming growth factor beta receptor 1(TGF-βR1) with potential antitumor activity mediated by reducing levels of active, phosphorylated SMAD [132]. A phase IB trial of this agent in combination with gemcitabine in advanced solid malignancies including pancreatic cancer demonstrated acceptable toxicity [133]. The PK profile of galunisertib was unchanged, and no DLTs were observed [133]. Galunisertib at 300 mg/day has been advanced into a randomized Phase II trial in pancreatic cancer in first-line setting to assess the antitumor activity of this combination [NCT02154646, NCT01373164].

### Talazoparib (BMN-673)

Talazoparib, also known as BMN-673, is a novel, orally bioavailable inhibitor of the nuclear enzyme poly (ADP-ribose) polymerase (PARP) with potent anti-neoplastic activity at subnanomolar concentration (IC_50_ = 0.58 nM) [134]. It selectively binds to PARP and prevents PARP-mediated DNA repair of single strand DNA breaks via the base-excision repair pathway. As a consequence, there is accumulation of DNA strand breaks, increased genomic instability, leading to apoptosis in target cells [134]. The agent has demonstrated efficacy with acceptable toxicity in patients with BRCA- mutant solid tumors including pancreatic cancer [135]. The recommended phase II dose is 1000 μg/day, and the main DLT is thrombocytopenia [135]. Additional phase I/II trials are ongoing [NCT02286687, NCT01989546, NCT01286987].

### Veliparib (ABT-888)

Veliparib (formerly ABT-888) is an orally bioavailable, carboxamide derivative with potent inhibitory property against PARP 1 and PARP 2 at concentrations (IC_50_) of 5.2 nM and 2.9 nM respectively [136]. Like talazoparib, ABT-888 selectively binds to PARP, inhibits DNA repair, and potentiates cytotoxicity of DNA-damaging agents such as alkylating compounds (e.g. temozolomide, platinums, cyclophosphamide) and ionizing radiation in syngeneic and xenograft tumor models [136, 137]. When combined with cisplatin and gemcitabine in a phase IB trial of untreated pancreatic cancer patients, veliparib demonstrated a tolerable toxicity profile with high clinical activity in BRCA-mutant tumors [138]. Serious adverse effects were anemia, neutropenia, thrombocytopenia, and fatigue. The recommended phase II dose was 80 mg PO BID on day 1–12 every 3 weeks with fixed doses of cisplatin (25 mg/m2 IV) and gemcitabine (600mg/m2 IV) on day three and ten [138]. Given these encouraging results, a two-part phase II trial of this compound has been initiated in this setting [NCT01585805]. In the first part, patients with untreated pancreatic cancer and BRCA and/or PALB2 mutations will be randomly assigned to receive gemcitabine and cisplatin with or without veliparib. In the other part, patients with relapses/refractory pancreatic cancer will receive veliparib alone. Patients from the first part whose cancer did not respond to gemcitabine and cisplatin treatment alone are eligible to participate in the second part of the trial.

### Salirasib (FTS)

The RAS signaling pathway plays a key role in signal transduction, cell motility, proliferation, survival and malignant transformation [17]. Up to 90% of human pancreatic cancers are driven by aberrant oncogenic RAS signaling and associated with increased tumor invasion and metastasis. Unfortunately, previous attempts to directly block RAS activity by inhibition of farnesyltransferase have been ineffective, as multiple escape pathways exist that allow for alternative prenylation of RAS protein [37, 139]. Salirasib is a novel, highly potent, synthetic small molecule with distinctive inhibitory property against RAS at micromolar concentration (IC_50_ = 2.6 μM) in kinase assays [140]. As a farnesylcysteine mimetic, salirasib selectively binds to and dislodges RAS from its membrane anchorage domains, and eventually accelerates its degradation and abrogates multiple RAS-dependent signaling pathways accompanied by substantial tumor growth inhibition in human pancreatic cancer xenografts [140].

The development of salirasib in advanced pancreatic cancer is still in the early clinical stage [141, 142]. A recently reported phase I study involving treatment-naïve patients with advanced, metastatic pancreatic cancer demonstrated good tolerability and encouraging clinical activity [141]. In combination with standard dose and schedule of gemcitabine, salirasib at 200–800 mg orally twice daily for 21 days every 28 days showed no overlapping pharmacokinetics. The most common adverse events were hematologic and GI toxicities, and fatigue [141]. The combination regimen achieved a median OS of 6.2 months and the 1-year survival of 37% in nineteen patients enrolled in the study [141]. The recommended dose for phase II trials was 600 mg twice daily.

## CONCLUSION

The survival of pancreatic cancer patients with either unresectable locally advanced or metastatic disease rarely surpasses 11 months with standard chemotherapy regimens. Recent efforts have identified multiple dysregulated signaling pathways in pancreatic cancer development and progression, many of which appear to be reasonable targets including PI3K/mTOR, SIRT1, and ALK [143–146]. The data from clinical trials on clivatuzumab, masitinib, veliparib, and salirasib are particularly encouraging. Further research into combinations of multiple targeted agents as well as mixture of these inhibitors in combination with cytotoxic agents to achieve optimal vertical or horizontal blockade will most likely improve the bleak outlook in patients with advanced/metastatic pancreatic cancer [147].
